# Likely country of origin in publications on randomised controlled trials and controlled clinical trials during the last 60 years

**DOI:** 10.1186/1745-6215-8-7

**Published:** 2007-02-27

**Authors:** Christian Gluud, Dimitrinka Nikolova

**Affiliations:** 1 The Cochrane Hepato-Biliary Group, The Copenhagen Trial Unit, Centre for Clinical Intervention Research, Rigshospitalet, Copenhagen University Hospital, 2100 Copenhagen, Denmark

## Abstract

**Background:**

The number of publications on clinical trials is unknown as well as the countries publishing most trial reports. To try to examine these questions we performed an ecological study.

**Methods:**

We searched the 454,449 records on publications in The Cochrane Central Register of Controlled Trials (CENTRAL) in The Cochrane Library, Issue 3, 2005 (CD-ROM version) for possible country of origin. We inspected a random sample of 906 records for information on country and type of trial.

**Results:**

There was an exponential growth of publications on randomised controlled trials and controlled clinical trials since 1946, but the growth seems to have seized since 2000. We identified the possible country of origin of 210,974 publications (46.4%). The USA is leading with about 46,789 publications followed by UK, Germany, Italy, the Netherlands, Canada, and France. Sweden becomes the leader with 891 publications per million inhabitants during the last 60 years followed by Denmark (n = 864), New Zealand (n = 791), Finland (n = 781), the Netherlands (n = 570), Switzerland (n = 547), and Norway (n = 543). In depth assessment of the random sample backed these findings.

**Conclusion:**

Many records lacked country of origin, even after the additional scrutiny. The number of publications on clinical trials increased exponentially until the turn of the century. Rather small, democratic, and wealthy countries take the lead when the number of publications on clinical trials is calculated based on million inhabitants. If all countries produced the same number of trials as these countries, this could mean thousands of new effective treatments during the next 60 years.

## Background

On the 20^th ^of May 1747, the Scottish naval surgeon James Lind started his controlled clinical trial of six interventions for scurvy patients [1]. The results were published in 1753 in his *Treatise of the Scurvy *[1]. Early examples of randomised, placebo-controlled trials date back to the Nuremberg salt trial from 1835 [2]. Physicians took their time. The whole idea of randomised controlled trials was first more widely accepted at the end of the Second World War [3]. Since then hundred thousands of clinical trials have been conducted all over the world [4,5], but many remain unpublished [6,7].

USA produced two-thirds of the scientific papers in the top 50 biomedical journals during 1995 to 2002, even after adjusting for population size, gross domestic product, and other factors [8]. These results confirm previous observations on the leading role of USA in clinical cardiology [9], clinical oncology [10], and biomedical research [11]. None of these studies – or other studies that we have been able to identify – have examined the production of randomised controlled trials and controlled clinical trials per country within all specialities.

The Cochrane Central Register of Controlled Trials (CENTRAL) in The Cochrane Library, being reputed as the most comprehensive trial register that exists [4,5], represents an opportunity to get an impression of the development over time and of the ranking of countries regarding publications on randomised controlled trials and controlled clinical trials. We aimed to assess the connection between number of inhabitants per country on one hand and publications on clinical trials on the other by the performance of an ecological study.

## Methods

We searched The Cochrane Central Register of Controlled Trials (CENTRAL) in The Cochrane Library Issue 3, 2005 (CD-ROM version) [4,5] to identify the number of publications on randomised controlled trials or controlled clinical trials per five-year interval and per country. The search terms were country names alone or a country name combined either with old or present names of the country and names of big provinces and big cities of each country. This was done since a separate search field tag with information for country of origin does not always exist in CENTRAL. We exported a random sample of 906 records (every 500 record) into a ProCite database for further analysis of the information on likely country of origin and type of trial. We identified country of origin by reading the record in CENTRAL. For the records without a country identifier, we searched EMBASE and MEDLINE via WinSPIRS, version 5.0, and Web of Science^® ^for country of origin. We sought the country of origin from the address field of those references that have one. For those without we sought country of origin through the town, city, province, or institution, so we could identify the likely country. Number of inhabitants per country in 2003 was extracted from the United Nations Department of Economic and Social Affairs [12]. We used Microsoft Excel to input data and produce charts ranking the top 20 countries.

## Results

**Figure 1:**
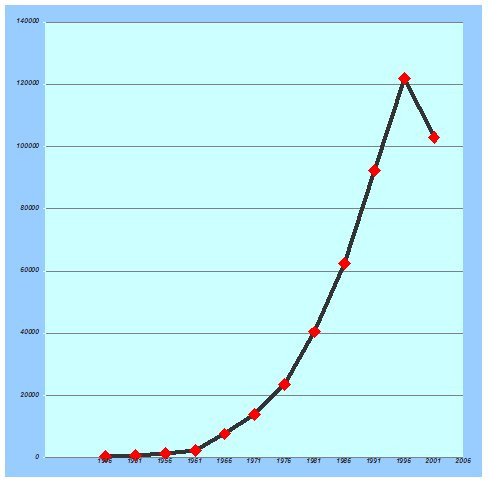
Number of publications on randomised controlled trials or controlled clinical trials per five-year interval since 1946 registered in CENTRAL in The Cochrane Library [4,5].

CENTRAL stored a total of 454,449 publications. The number of publications per five-year period after 1946 is shown in Figure 1. The number of publications increased exponentially to 2000. The growth seems to have seized at the turn of the century. During the last 10 years about 25,000 publications on randomised controlled or controlled clinical trials have been published each year.

**Figure 2:**
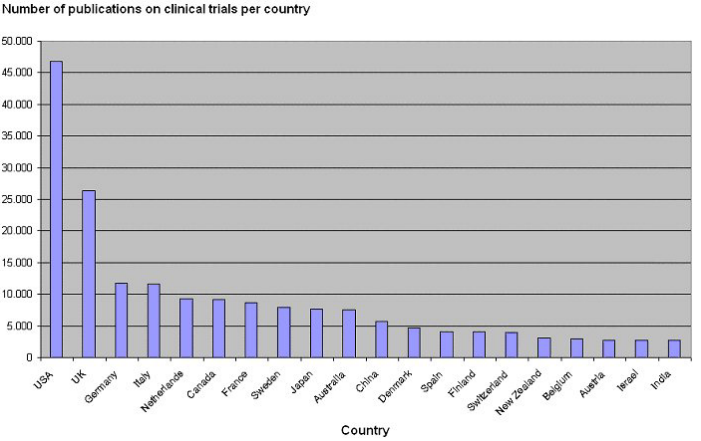
Number of publications on randomised controlled trials or controlled clinical trials published per country since 1946 registered in CENTRAL in The Cochrane Library [4,5].

We could identify the possible country of origin of 210,974 publications in CENTRAL (46.4%). USA is the country producing most publications on randomised controlled trials or controlled clinical trials during the last 60 years (n = 46,789; 22.2%), followed by UK (n = 26,401; 12.5%), Germany (n = 11,782, 5.6%), Italy (n = 11,587; 5.5%), the Netherlands (n = 9,233; 4.4%), Canada (n = 9,134; 4.3%), and France (n = 8,666; 4.1%) (Figure 2).

We inspected the contents of the randomly selected 906 records from CENTRAL. Our observations showed that 419 records (46.2%) were tagged as randomised controlled trials by MEDLINE; 209 records (23.1%) were tagged as randomised controlled trials by the Cochrane Review Group that had submitted the record for inclusion in CENTRAL; and 193 records (21.3%) were controlled clinical trials either tagged by MEDLINE or the Cochrane Review Group. Accordingly, 821/906 references (90.6%; 95% confidence interval 88.5 to 92.4%) were references to randomised controlled trials or controlled clinical trials. The majority of the records being clinical trials were randomised controlled trials (628/821; 76.5%).

Only 368 records out of the 821 records on randomised controlled trials or controlled clinical trials gave a country identifier (44.8%, 95% confidence interval 41.4 to 48.3%). Only 29 records out of the 85 records, which did not refer to a randomised controlled trial or a controlled clinical trial, gave a country identifier (34.2%; 95% confidence interval 24.2 to 45.2%). We searched EMBASE and MEDLINE via WinSPIRS, version 5.0, and Web of Science^® ^to identify the likely country of origin of the remaining 509 references without a country identifier. We were able to identify a likely country of origin for an additional 163 of 453 references on randomised controlled trials or controlled clinical trials and 14 out of 56 references on records, which did not seem to deal with a clinical trial. The distribution of countries between the 368 references with a country of origin and the 163 references without a country identifier in CENTRAL was not significantly different (data not shown). Most of the records in CENTRAL for which we were unable to identify a likely country of origin were references to meeting abstracts.

**Figure 3:**
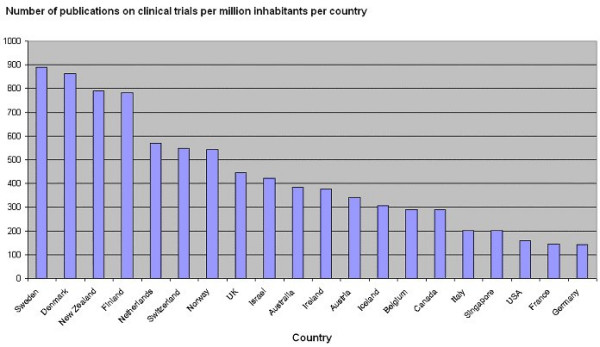
Number of publications on randomised controlled trials or controlled clinical trials published per million inhabitants per country since 1946 registered in CENTRAL in The Cochrane Library [4,5].

We calculated the number of publications on randomised controlled trials and controlled clinical trials per million inhabitants in each country during the last 60 years (Figure 3). Ranked in this way, Sweden (n = 891), Denmark (n = 864), New Zealand (n = 791), Finland (n = 781), the Netherlands (n = 570), Switzerland (n = 547), and Norway (n = 543) are the countries having produced the largest number of publications on clinical trials per million inhabitants during the last 60 years. In comparison, UK only published about 445 and U.S.A. only about 159 publications on clinical trials per million inhabitants during the last 60 years.

## Discussion

The major findings of our ecological study are that the number of publications on clinical trials has grown dramatically since 1946. The growth seems to have seized at the turn of the century. If the indicator for comparison is total number of publications, then unsurprisingly USA is first. The ranking of countries dramatically change when one compares the number of publications per country with the number of publications per million inhabitants per country during the last 60 years. Then rather small, democratic, and wealthy countries take the lead.

Our study has a major advantage: we searched CENTRAL in The Cochrane Library. CENTRAL represents the largest collection of citations to publications of randomised controlled trials and controlled clinical trials in the world [4,5]. CENTRAL has identified the records on clinical trials through systematic searches in MEDLINE (covering mainly North American and European journals), EMBASE (covering mainly European journals), Australasian Medical Index, Chinese Biomedical Literature Database, and LILACS (Latin American Caribbean Health Sciences Literature) [4,5]. In addition, each of the 50 Cochrane Review Groups of The Cochrane Collaboration have supplied extensive handsearch results for CENTRAL [4,5]. Therefore, CENTRAL includes citations to randomised controlled and controlled clinical trials not indexed in MEDLINE, EMBASE, or other bibliographic databases. Such citations refer to trials published internationally in many languages and to trials only published in conference proceedings or other hard-to-access sources or unpublished trials [4,5]. Such publications are important because only 63% of abstracts on randomised controlled clinical trials are later published as full text papers [13]. Furthermore, CENTRAL is a quickly expanding database. The groups submitting records for inclusion in CENTRAL revise and update the publication type code after inspection of the full text of the publication or through communication with any of the authors. This may lead to discrepancy of tagging of Cochrane records when compared to records obtained from MEDLINE or other databases. During the process of collecting records from countries like China or Japan, many publications on trials that state with one word to be randomised controlled or controlled, but in fact are not, could have entered CENTRAL. However, wrongly included records are discovered with the production of Cochrane systematic reviews since CENTRAL is their main source of evidence. We were positively surprised that about 90% of the records in CENTRAL referred to publications on randomised controlled trials or controlled clinical trials. This finding lends strengths to our findings.

Our study has a number of potential shortcomings. We would have reached other results if we had searched other databases or collections of databases or if all trials were with a tag for country. Had we searched, eg, PubMed (which includes MEDLINE plus citations not added to MEDLINE yet) using the limits 'randomised controlled trial' and 'human' we would have obtained only 209,909 citations, of which 38,359 referred to USA (18.3%), 11,073 to UK (5.3%), 1361 to Canada (0.6%), 1099 to Germany (0.5%), 890 to Italy (0.4%), and 889 to The Netherlands (0.4%) (date of search 14 April 2006). Accordingly, our figures would have changed – in some instances dramatically, in other instances less so. The ranking of countries would also have changed, but apparently not dramatically. Our figures should be taken with a grain of salt. They are not representing any true number. They should be interpreted as relative figures for comparison among countries.

Second, we could only attribute a likely country of origin to about half of the publications in CENTRAL. Inspecting the random sample of 906 records for possible country of origin confirmed this observation [see Additional file 1]. The difference between the two ways of searches was minor, ie, 1.6%. The figures we have obtained are rough estimates, more reflecting relative country contributions than actual numbers. However, this may not represent a major problem unless somebody can identify a non-random process that determines which records achieve a country tag and which do not. By additional searchers in EMBASE and MEDLINE via WinSPIRS, version 5.0, and Web of Science^® ^we were able to identify a likely country of origin for an additional 163 records on trials in CENTRAL. The country distribution among these 163 records did not differ significantly from the distribution among the 368 records with a country identifier. This suggests that a random process has selected which record has a country identifier and which has not.

Third, about 10% of the random sample of 906 records could have been included in CENTRAL in error. This percentage is low and lessens the concern of false positive records on randomised controlled trials and controlled trials when related to identifying their possible country of origin. We have, therefore, disregarded this number in our calculations, as we see no reason why the proportion of non-controlled studies in CENTRAL should be country dependent.

Fourth, we have been unable to account for the impact of doublets and other misclassifications. However, they are minor and not likely to be able to change the ranking of countries dramatically.

Fifth, CENTRAL suffers from the facts that it mainly includes clinical trials from MEDLINE and EMBASE, and that it does not include all clinical trials from all topic-specific or country-specific registers in the World. There are a number of trial registers and hence trials from countries that are not included or fully represented when one searches CENTRAL [14-16]. This is important to remember, but it is not likely to change the ranking of countries in the top 20 dramatically. Furthermore, a number of, e.g., Chinese studies turn out not to be properly randomised [17,18]. The consequences of including registers from such countries should accordingly be examined in depth and require other study designs that we have presently embarked on.

Sixth, CENTRAL is not a study-based register. Thus, we had no chance to take into account multiple publications of the same trial. Each randomised controlled trial is published about twice [19]. As stated, country of origin of the trials is not contained in the published reference, which makes it difficult to identify the origin of more than 50% of the published trials. Furthermore, we do not know if the country linkage we identified represents the country co-ordinating the trial, the country in which the participants were included, or the country in which the trial was published (e.g., in case of abstracts and meeting proceedings). Again, such misclassifications are not likely to dramatically change the ranking of countries.

Seventh, our study suffers from publication bias, ie, the fact that trials are conducted, but never published [6,7]. Numerous studies show that only 30% to 80% of conducted trials are published [20-23]. During the last 10 years about 25,000 publications on clinical trials are published per year according to CENTRAL. These figures translate into about 500 trials being launched each week if each trial is on average published twice and only 50% of the trials are ever published. This number of trials is double the average number of 250 clinical trials registered per week with ClinicalTrials.gov in USA from all over the World [24]. This indicates that registration is not yet 100%, registrants may use other registries, or the speed of trial launch has decreased more dramatically than our data suggest.

The underreporting of trials is a serious ethical problem, which undermines the confidence of people in the health-care systems [6,7,20-23]. Furthermore, underreporting may cause health hazards. It is, therefore, important that the International Committee of Medical Journal Editors [25], the World Health Organization (WHO) [26], and others [27] endeavour at getting trial registration implemented worldwide. The WHO decided in May 2006 to urge researchers and companies to register all medical studies that test treatments on human beings, including the earliest studies, with patients or healthy volunteers [28,29]. WHO's International Clinical Trials Registry Platform will standardise the way information on medical studies is made available to the public. WHO recommends that 20 key details be disclosed before inclusion of the first trial participant. These details include the country of origin of the sponsor of the trial, of the investigators, as well as of the participants of the trial. The fact that we were only able to identify a likely country of origin of about 46% of the records in CENTRAL underlines the urgency with which we have to implement WHO's call for trial registration. This call must be implemented in national laws. The Registry Platform is not a register itself, but it provides a set of standards for all registers that can be searched via the Registry Platform. WHO has not only standardised what must be reported to register a trial, but is creating a global trial identification system that will confer a unique reference number on every qualified trial. As indicated above, registration has to increase substantially before full coverage is secured.

During the last 60 years a number of countries have managed to publish almost 700 publications on trials per million inhabitants without their patient groups, their populations, or their health-care systems to have been overburdened by clinical trials. In fact, some of the countries with most publications on trials seem to have a more positive attitude to clinical trial activity. If only 50% of trials are published and those published are published about twice, the publication figure translates into the conduct of about 700 trials per million inhabitants during the last 60 years. This figure is a low estimate for current conduct of trials. Using the figure of 700 trials per million inhabitants as a 'benchmark' for all countries, the next 60 years could bring us about 4.5 million new trials. For medicine, this will mean a great leap forward. If just 1% of the tested interventions are to show clinical benefit, this could bring 7,500 new effective treatments to the market if the count continues to be about six trials per intervention [19,30].

The drop in number of publications during the last five-year interval is likely due to a combination of factors, like backlog in getting trial publications registered in CENTRAL, slow down in the pace of clinical development, and potential negative influence of the EU Directive 2001/20 on conduct of randomised controlled clinical trials [31]. Our findings of a decrease are confirmed by other reports, showing that the problem is not only European [32].

The vast majority of published controlled trials have been conducted with insufficient sample sizes and inadequate methodologies [33-40]. There is a growing understanding in the world to strengthen and facilitate randomised controlled trials [41-44]. The European Clinical Research Infrastructures Network (ECRIN) has created The International Clinical Trials' Day each year on the day James Lind started his famous trial: 20th of May [1,28,44]. Hereby, we hope to increase the public understanding worldwide of the importance of clinical research to health care. In 2006, The International Clinical Trials' Day was marked in Brussels by an ECRIN workshop and press conference supported by EU and the WHO [28].

Due to the above limitations of our study, we find that further analyses of the number of publications on clinical trials per country are warranted. Such studies could look at the numbers based on other literature databases as well as WHO's Registry Platform. Furthermore, studies could analyse the clinical trial activity by taking into consideration educational levels, gross domestic product, morbidity, mortality, and other variables.

## Competing interests

None known. CG has been working actively in ECRIN, WHO, and the Ottawa Group for getting clinical trials registered and marketed around the World by introducing The International Clinical Trials' Day on 20th of May. CG and DN are supporting the Cochrane Collaboration.

## Contributions of authors

CG originated the idea, performed searches, and drafted the manuscript. DN performed searches, read all 906 CENTRAL records, performed calculations, and drafted the figures and part of the manuscript. Both authors have read and accepted the final version.

## Supplementary Material

Additional file 1 Identified country and type of trial of the 906 randomly selected records. The data provided represent the findings of an in-depth scrutiny of a random sample of 906 records. Click here for file
