# Supplementary material for: Likely country of origin in publications on randomised controlled trials and controlled clinical trials during the last 60 years
Source: Trials. 2007 Feb 27;8:7. doi: 10.1186/1745-6215-8-7 (PMC1808475; doi:10.1186/1745-6215-8-7)
Supplement: Additional file 1 — Identified country and type of trial of the 906 randomly selected records. The data provided represent the findings of an in-depth scrutiny of a random sample of 906 records. [file 1745-6215-8-7-S1.doc]

|  | **RCT or CCT (90%)** | | Not CCTs or not RCTs or no tagging | | |
| --- | --- | --- | --- | --- | --- |
|  | With a country identifier | Without a country identifier | With a country identifier | | Without a country identifier |
|  |  | **290** |  | | **42** |
| USA | 107 | 64 | 6 | | 7 |
| UK | 40 | 11 | 2 | | 0 |
| Germany | 26 | 11 | 3 | | 1 |
| Italy | 19 | 16 | 2 | | 1 |
| France | 16 | 4 | 3 | | 1 |
| Netherlands | 14 | 6 | 2 | | 0 |
| China | 14 | 2 | 0 | | 0 |
| Denmark | 13 | 5 | 2 | | 1 |
| Sweden | 11 | 4 | 2 | | 0 |
| Canada | 10 | 4 | 0 | | 1 |
| Australia | 10 | 3 | 0 | | 0 |
| Belgium | 10 | 0 | 0 | | 0 |
| Japan | 8 | 7 | 1 | | 0 |
| Spain | 7 | 3 | 0 | | 0 |
| Switzerland | 5 | 3 | 0 | | 0 |
| Finland | 5 | 1 | 0 | | 0 |
| Austria | 4 | 3 | 2 | | 0 |
| India | 3 | 2 | 0 | | 0 |
| New Zealand | 0 | 0 | 0 | | 1 |
| Norway | 0 | 4 | 0 | | 0 |
| Israel | 0 | 1 | 0 | | 0 |
|  | | | | | |
| **OTHER COUNTRIES** | | | | | |
|  | | | | | |
| Turkey | 8 | 2 | 1 | 0 | |
| Poland | 6 | 0 | 0 | 0 | |
| South Africa | 2 | 1 | 0 | 0 | |
| Taiwan | 2 | 1 | 1 | 0 | |
| Brazil | 2 | 0 | 1 | 0 | |
| Hungary | 2 | 0 | 0 | 0 | |
| Greece | 2 | 0 | 0 | 1 | |
| Iran | 2 | 0 | 0 | 0 | |
| Nigeria | 2 | 0 | 0 | 0 | |
| Singapore | 2 | 0 | 0 | 0 | |
| Egypt | 1 | 1 | 0 | 0 | |
| Slovak republic | 1 | 0 | 0 | 0 | |
| Pakistan | 1 | 0 | 0 | 0 | |
| Zambia | 1 | 0 | 0 | 0 | |
| Venezuela | 1 | 0 | 0 | 0 | |
| Bangladesh | 1 | 0 | 0 | 0 | |
| UAE | 1 | 0 | 0 | 0 | |
| Botswana | 1 | 0 | 0 | 0 | |
| Indonesia | 1 | 0 | 0 | 0 | |
| Saudi Arabia | 1 | 0 | 0 | 0 | |
| Korea | 1 | 0 | 0 | 0 | |
| Mexico | 1 | 0 | 0 | 0 | |
| Kenya | 1 | 0 | 0 | 0 | |
| Czech Republic | 1 | 0 | 0 | 0 | |
| Malaysia | 1 | 0 | 0 | 0 | |
| Thailand | 1 | 0 | 0 | 0 | |
| Columbia | 0 | 1 | 0 | 0 | |
| Nairobi | 0 | 1 | 1 | 0 | |
| Morocco | 0 | 1 | 0 | 0 | |
| Ethiopia | 0 | 1 | 0 | 0 | |
|  |  |  |  |  | |
| **In total = 906** | **(368 +** | **453)** | **(29 +** | **56)** | |
